# The Hospital Officer's Bookshelf

**Published:** 1913-02-15

**Authors:** 


### THE HOSPITAL OFFICER'S BOOKSHELF.

### AN OPERATING THEATRE IN PRIVATE PRACTICE.

By C. Hamilton Whiteford, M.R.C.S., L.R.C.P. (London: Harrison and Sons, 45 Pall Mall, S.W. 1912.)

Dr. Whiteford is well advised to lay special stress on the prime importance of insisting on the essentials of perfect asepsis in all surgical work, and his little book is an admirable compilation of what are the requisites for securing such perfection in the operating room. To those interested in hospital construction, no less than to the practitioner who wishes to get hints with regard to the building and equipping of his private theatre, the book should prove of service, for it gives several good descriptions, and, what is perhaps more important, some interesting suggestions. Dr. Whiteford's aim, as he expresses it, has been "to evolve a building in which strict asepsis can be observed with a minimum of labour for all concerned." He proceeds to give a sketch of what this ideal is like in practice. We find that the nursing home to which the theatre is attached is a long building with only a ground and first floors; the theatre unit forms a wing on the first-floor level, shut off from the rest of the building by folding doors. These doors give access to a lobby leading into the anæsthetist's room, beyond which is the theatre itself. A dressing and a sterilising room are also provided, opening out of the lobby. Some interesting notes are given with regard to the materials. The floors are of cement, which is much cheaper than more expensive but perhaps less absorbent flooring; all piping is hidden, being covered in by detachable metal or uralite plates. Glass has been reduced to a minimum, and brass has been avoided in the fittings on account of the difficulty of keeping such fittings polished and clean; aluminium and plated brass fittings or painted iron fittings have been used instead. The windows and doors are of simple pattern. Some interesting details are given with regard to the heating. This is by an electric heater sunk into the wall six inches above the floor level. The plate measures six feet by six inches. There are four such heaters in the theatre and two in the anæsthetising room. Dr. Whiteford estimates the cost of running each heater at a little over a penny per hour. This seems to us a high price to pay for heating, but the arrangement has the advantage of being almost dustproof and of economising labour. Whether the plates will wear well remains to be seen; our own experience of electric-heating apparatus for theatre use has been very disappointing, and we should hesitate to recommend the use of such contrivances in preference to low-pressure hot-water pipes. The theatre itself is 13½ feet high, with 14 by 14 feet of floor space, which gives ample room for most ordinary work. A south, rather than the usual north, aspect has been deliberately chosen. "We find," remarks Dr. Whiteford, "the south aspect to give such excellent light that, even when operating in deep cavities, artificial illumination has rarely been found necessary. . . . A minor advantage is that the south aspect, by affording natural warmth, minimises the amount of heat required. . . . We have operated in this theatre during extreme degrees of heat and cold in the outdoor temperature, and find no cause to regret the choice of the southern aspect." This opinion is interesting, though we cannot say that the arguments appeal to us very strongly, since a northern aspect has equal advantages, to say the least, as far as light is concerned, while in an efficiently heated theatre the question of utilising that problematical addition of sun-heat due to a southern aspect is hardly likely to be a matter for serious consideration. During the daytime the room is lighted by a large plate-glass window of simple construction in the south wall; artificial lighting is provided for by a quadruple set of lamps attached to the ceiling and so arranged that no shadows hamper the operator. Dr. Whiteford prefers to work in an atmosphere of not higher than 65° Fahrenheit, which seems to us rather low for a fully anæsthetised patient, although we frankly admit that the fashion of over-heating theatres, against which he warns us, is responsible for much of the discomfort that results to patient and surgeon alike.

A full description is given of the ventilation of the theatre and of the equipment. This description, while presenting nothing new to those who are familiar with the details of theatre construction, is instructive enough to those who are not fully acquainted with such details, and deserves careful reading. In fact the little book abounds in suggestions, the majority of which are the outcome of the writer's own experience. To the expert surgeon many of the remarks made may appear trite and commonplace, but we think Dr. Whiteford does well to insist on these commonplaces, for they enshrine golden truths which should be constantly before the surgeon who operates, whether he does so in a large general hospital or whether he works in a small private room.

### A MANUAL OF SURGICAL TREATMENT.

By Sir W. Watson Cheyne, Bart., C.B., F.R.S., and F. F. Burghard, M.S., F.R.C.S. New Edition, revised and rewritten with the assistance of T. P. Legg, M.S., F.R.C.S., and Arthur Edmunds, M.S., F.R.C.S. Vol. III. (London: Longmans and Co. Pp. 575. Price 21s. net.)

This third volume of the five which are to complete the new series of Cheyne and Burghard bears ample evidence of the painstaking care and thoroughness with which the revision of the original has been carried out. It deals with the treatment of surgical affections of the joints, the spine, the head, and the face. The first chapter plunges at once into the treatment of simple traumatic dislocations, and gives a most valuable account of the subject. The descriptions and diagrams by which the necessary manipulations are explained are most lucid, and emphasis is very properly laid upon several small details in the various procedures which are often overlooked but are really essential to successful results. The account of the celebrated Kocher's method of reducing dislocations of the shoulder is a good instance in point; it differs in several respects from the accepted procedure as taught by most British authors, and is an exact account of what the Berne surgeon actually practises. In passing, we may note that the authors recommend that an anæsthetic should always be administered before any attempt is made to reduce a dislocation. This contention seems to us to be wholly untenable. To begin with, the majority of those who sustain dislocations have partaken of food—often of a "square meal"—within the previous five hours. They are thus by no means ideal subjects for anæsthesia; so the only alternative (according to the authors) to subjecting the patient to a definite risk would be to let him wait a very considerable time before any attempt is made to relieve his injuries. During this waiting period muscular spasm and articular swelling are more likely to increase than to diminish, and so to render the reposition of the dislocated bones more difficult than ever. Then, too, the reduction of a dislocation is not by any means an invariably painful process. Every practitioner must have seen dislocations reduced so readily that the patients have been surprised to know that anything was being undertaken for their relief. It may be granted that in most cases the reduction of dislocations without anæsthesia is painful and difficult; but that is no reason why a tentative effort should not be made to manipulate the bones into apposition, for if it proves to be painful the attempt can easily be abandoned with no harm done. Apart from this one small point, we have nothing but praise for the section upon dislocations; if we were asked to single out one case for especial commendation, the pages upon the thumb would perhaps obtain our suffrage. Wounds and inflammatory affections of joints are also admirably described; but four pages is too measly an allowance for sprains, and this chapter ought to be considerably expanded. The chapters on diseases of the individual joints are also excellent, and thoroughly practical everywhere.

In the treatment of cleft palate it is natural enough, in view of Mr. Legg's close association with Mr. Berry over this topic, that the Langenbeck operation as performed by that surgeon should be awarded pride of place. The rival operation of Arbuthnot Lane is falling out of favour amongst surgeons, probably with good reason; the Manual does not overstate the case for the preference expressed, and deals fairly also with the other methods which have been advocated for the remedy of this deformity. Another useful section is that in which Dr. d'Este Emery reviews the uses of lumbar puncture in injuries and diseases of the nervous system and of the meninges. Taken all round, we feel inclined to say that this volume is the best of the three so far published. This is praise indeed, for the first two volumes attain a very high standard; but it is not undeserved, and if the next two volumes are equally good the completed work will reflect much honour upon British surgery.

### THE TREATMENT OF TUBERCULOSIS.

By Walter Fearis. With Introduction by Dr. Carl Spengler. (London: John Murray.)

As much truth as the Heraclitean principle will allow in anything lies in the old saying that a man should be judged by his peers. Good observers, whatever their cast of temperament, all agree as to this, although they fail sometimes to act up to it. For an illustration from medical life let us go back in memory to those somewhat receded days when we were second-year medical students; when, as the late Sir William Broadbent remarked with the candour his face expressed so thoroughly, and as Dickens intimated in the artist's way in his picture of Bob Sawyer, we had decidedly exaggerated ideas of the eminence of the teachers we found presiding over our curriculum of studies. In the ardour of youth for new things and a change from home surroundings we would bore our friends a little with rhapsodies of admiration, disillusionment only coming gradually with fuller knowledge of the world. Walpole Cavendish, we would confide, didn't know much, was no good, in fact, although a good sort, and in his time a cricket Blue; but Harley Wimpole—ah! tremendously "thick"!—knew more about nervous diseases than anybody; whilst old Welbeck was the best man going on fractures. And so on, until at last some senior relative gently told us that, although our strong feeling of solidarity did us credit, yet, after all, it was remarkable that all these gentlemen had the honour to belong to St. Ursula's, where we, by a strange coincidence, had entered for our studies. Suppose, they went on in effect, you had gone instead to those equally famous schools, St. Wilfred's or the Metropolitan, might you not have found paragons and nonpareils there too?

For this pleasant little recollection must be thanked Mr. W. H. Fearis, formerly demonstrator of botany at University College, Reading, who, during a stay at Davos, has been so much taken with the medical work of Dr. Carl Spengler as to write a short book on it. Dr. Spengler's name does, of course, stand high. Nearly every well-informed medical man knows of his idea of using bovine tuberculin for disease caused by the human type of bacillus. The great Koch entrusted much to him; recently he has said that the source of protective substances in tuberculosis is the red corpuscles of the blood, and has put forward on the base of this theory an agent, IK, for producing passive immunity. Mr. Fearis, out of the fulness of his unacquaintance with modern medical research, proclaims all these undertakings and more also not as work on its trial but as gospel truth rather unfairly neglected; and we can only tell him what our fathers have told us. If, we would venture to ask, you had gone, not to Spengler at Davos, but to Denys at Louvain, or to Petruschky at Danzig, Mitulescu at Bukharest, Wright in London, Maragliano in Italy, or to somebody else somewhere else, might you not have been propagandising just as fervently to another tune altogether? Truth to tell, something like this seems to have struck Dr. Spengler himself, for in his short introduction he mentions that it was contributed at the author's request, and ends up by saying that his wish is that the book may aid in lightening the burden of the suffering tuberculous; as much as to discount its certain result of a "boom" of himself by its greater result of furthering therapeutics, which latter effect he, of course, believes to be as certain as the former one. We might, therefore, supplement the excellent parental argument by playing the always easy *rôle* of devil's advocate, as follows.

Not even a man of Dr. Spengler's scientific calibre is above subconscious mental bias. Now he says that the erythrocytes are what produce protective substances in tuberculosis. But he works at Davos, which is in the mountains; and certainly erythrocythæmia is an effect of high altitude. Do all workers, or even most workers, use in consumption P.T.O. instead of human tuberculin? By no means. Is the balance of opinion among them in favour of IK? No, it is not. The thirteenth chapter of the book contains an analysis of reports on the remedy by other physicians, of whom 65 per cent. are stated to be favourable to it. But this analysis is incomplete, as is shown by the following test. In the leading *Centralblatt* on tuberculosis there are, from the beginning of last year to the present date, mentioned ten papers on IK. Of these only four are favourable, and one of them, as the reviewer significantly notes, recommends inunction of a few drops of the third dilution of IK for infants with the stomach ache, a decidedly mean attempt at ousting the humble but useful dill water. The other three (Benöhr, Autokratow, Lukin) Mr. Fearis duly mentions. Of the six unfavourable reports—Starkloff, Baer, Benker, Karpilowsky, Kascherininowa, Galecki, and Budrynski—he gives only Karpilowsky, whose paper was by no means the earliest published. We impute no bad faith to anyone. It is merely the necessary want of experience by a layman in dealing with a particularly crowded section of the huge subject of medicine. Nor do we particularly grudge Switzerland this ill-informed *réclame*. Rich consumptives will always continue to go there, both because the winter surroundings are more pleasant than in this country, and because there is less chance of juxtaposition of their social inferiors; not merely from the view that consumption curing is "one of those things they do better abroad." As has been seen, there is a large choice available of foreign specialists.

Vicious Circles in Disease. By Jamieson B. Hurry, M.A., M.D. (Cantab.). Second and Enlarged Edition. (London: J. and A. Churchill. Pp. 262. Price 7s. 6d. net.)

By a vicious circle is meant a condition in which two or more disorders are so correlated that they reciprocally aggravate and perpetuate each other. That is the foundation-stone of Dr. Hurry's whole argument; and it was laid, he considers, by Asclepiades about a century before Christ. Every student of medicine is familiar with instances of such vicious circles, and all his text-books and teachers refer to instances of them. In Dr. Hurry's view these references are not sufficiently emphasised to give the student and the young practitioner a full conception of the frequency and the importance of the vicious circle in the incidence and treatment of disease; so he has undertaken to collect and exemplify them by devoting an entire monograph to the subject. As a monument of industry the result reflects great credit upon its author, who unquestionably leaves this thorny and difficult question materially more clarified than he found it. His acquaintance with medical literature is exhaustive, and on every page proofs abound that only a man of exceptional catholicity and culture could have tackled the matter as he has done. Nor does he fall into the pit that so many medical pioneers seem to dig for themselves, of blindly ascribing every conceivable malady to a pet hypothesis. Thus, in the chapter treating of eye diseases and vicious circles in connection therewith, we find him recognising very frankly that with improving knowledge some processes once thought to be viticircular (if that is the right adjectival form) in nature are in reality proved to be nothing of the kind. In his sections upon breaking the circle his outlook appears to us somewhat less philosophic; for he attributes various phenomena of disease to Nature's efforts to break vicious circles, which for our own part we should prefer to regard as cases where no such circles exist. At all events, Dr. Hurry has made his colleagues in the medical profession think; that achievement is not an easy one, and we congratulate him on it. The enlarged second edition of his book is evidence both of the success of the first edition and of his unabated energy in thrashing out the topic. Would he be disposed to accept a suggestion that the third edition, when it is wanted, should be somewhat less eccentric in the matter of type?

Walt Whitman's Anomaly. By Dr. W. C. Rivers. (London: George Allen and Co., Ltd. 2s. 6d. net.)

It is surely a little late in the day to write a brochure to prove that Walt Whitman was homosexual. Still, if Dr. Rivers tells a twice-told tale, he tells it with care, knowledge, and distinction; but we are surprised that one whose diction is so fragrant of the study should have been thrown off the scent by Whitman's indignant denial of certain questions which J. A. Symonds put categorically to him. The poet is the best critic of other men's work, but in the deepest aspect of criticism generally the worst of his own. Consequently the only, and luckily the shortest, way to understand Walt Whitman's temperament is to read his poems. Their purport is indisputable, as the author profoundly explains. Having chosen a less direct method, Dr. Rivers may perhaps pardon us for thinking that he seems rather too forcibly to assume that genius is the reverse side of degeneracy. It is easy to show, of course, that "men abnormal in favourable ways are also abnormal in unfavourable ways"; but then men normal in favourable ways are also normal in unfavourable ways. In short, the assumption lying at the base of this argument is that of the normal man. He does not exist any more than his ill-fated economic brother. The most normal healthy man in the world, to an acute observer who has an interest in obtaining the necessary evidence, presents innumerable stigmata. The homogenic temperament is one which every individual possesses *in posse*, which many find an active source of heart-searchings at or before the age of puberty, which a few preserve undiverted to the end of their lives. But, to avoid misapprehension, we must ask readers not to confuse the very sharp distinction that exists between the temperament and certain criminal actions. In the public idea the one is a synonym of the other. In practice the distinction must be sharply drawn. Apart from one passage where Dr. Rivers notes this, he seems not always to dissociate them. In fine, a writer combining this author's culture and medical training is too rare to be wasted, hence our careful attempt to do justice to his admirably planned and well-written work.

Massage and Swedish Gymnastics. By Thomas Luke, M.D., F.R.C.S. (London: The Scientific Press, Ltd., 28 and 29 Southampton Street, Strand, W.C. Price 2s. 6d. net.)

Dr. Luke's little manual of Swedish gymnastics and massage is an admirable book for the beginner. It gives details of the movements and manipulations in simple and clear language, and contains many useful hints with regard to actual treatment. We are glad to find that Dr. Luke lays stress on certain essential points. One of these is the necessity for a thorough mastery of detail. As the author justly remarks, in cases of fracture, any nurse who has been trained to give massage gently and soberly is qualified to undertake the simple manipulative after-treatment; this demands no high degree of expert skill, but it does demand gentleness and the ability to discriminate. The special technique of massage is described with commendable brevity; here and there, perhaps, the descriptions are too clear cut; but, on the other hand, it must never be forgotten that massage cannot be learnt from books. All that this little work attempts is to give the nurse or students an outline of certain actions, movements, and manipulations, which, in order to be fully grasped, should be seen and assiduously practised before the masseur or masseuse commences work on his or her own account. As such the book is very valuable; it is the shortest and clearest little manual on the subject we are acquainted with, and it is strikingly free from the faults which one finds very often in larger and more ambitious works. The section dealing with medical gymnastics is especially good. The line blocks and diagrammatic illustrations elucidate the text and really help the student. Frenkel's exercises for tabes are well described, and sections are devoted to some other special forms of remedial exercise. The chapter on spinal curvature is short, and not so well written as the other sections. It should be mentioned that scoliosis is associated in many cases with flat feet, and a note on the Klapp method of treatment by crawl exercises will be added, no doubt, in the later editions that are sure to be required. As to the last chapter in connection with the treatment of flat foot—the student must learn that no one type of manipulations can be generally used, but that each case must be studied from its individual anatomical peculiarities.

Manual of Medicine. By A. S. Woodwark, M.D., M.R.C.P. (London: Henry Frowde and Hodder and Stoughton. 1912. Pp. 409. Price 10s. 6d. net.)

We can readily understand that some of the clinical teachers of the day, especially those whose medical education was completed in days of less hustle and bustle than the present, may shake their heads over this text-book, which aims at providing medical students with the essential outlines of medical science in the most compact and easily remembered form. Yet for fourth and fifth year students it will prove, we honestly believe, a most valuable compilation; for it gives them something that they will appreciate and absorb, notwithstanding the (possible) disapprobation of reverend senior physicians. It would be a great pity if any medical student tried to make Dr. Woodwark's book a substitute either for clinical study in wards and out-patient rooms, or for the subsequent absorption of larger and fuller manuals of medicine. But there are two states of student existence in which it has a value of its own. One is the early stage of clinical clerking, when the receptive mind of the student is apt to be appalled by the immensity and abstruseness of the tomes in which he is usually recommended to seek a knowledge of medicine. The other is that of the man who is shortly to "sit" for his final and wants a rapid general survey of his subject—orderly, trustworthy, and well balanced. Probably a student could "get through medicine" without reading any other text-book than this, provided he had worked hard in the wards; but we sincerely hope none will try to do so, for that is as much outside the author's intention as it is contrary to his readers' real interests. In a word, this is the best "cram" book we have ever come across; but it is not, and does not pretend to be, the whole science of medicine.

### Dermatology.

A new text-book on the Diseases of the Skin is announced by Messrs. Baillière, Tindall and Cox as being nearly ready for publication. The author is Dr. David Walsh, senior physician to the Western Skin Hospital, and the book is primarily intended for students and general practitioners. It is not too bulky a volume, and at the popular price of five shillings or thereabouts should command a good sale. Dr. Walsh, it may be recalled, has done a good deal of special work during the last few years tending to show that faults in the circulation, whether in the heart or in the vessels, have a very important influence in the causation and the course of chronic or recurrent diseases of the skin. Much of this work which was outlined in a paper read at the last meeting of the British Medical Association will be found incorporated in this latest text-book, together with the advances that have been made in the bacteriology, etiology, pathology, and treatment of the diseases of the skin.

### "The Prescriber" for 1912.

For a monthly journal dealing with therapeutics and treatment the seventh volume of *The Prescriber* deserves to be more widely known than most bound newspapers since it contains a very complete and practical record of therapeutic progress, besides able articles of medical interest. One of its good features is the publication quarterly of a therapeutic summary and index to current literature, which will be found most useful for reference. Skimming through the bound volume for 1912 one's attention is continually arrested by interesting notes on the very latest pharmaceutical preparations or methods. In the current number there are published a number of typical prescriptions for use by panel doctors under the National Insurance Act. The ingredients are all to be found in the official list, and these prescriptions may be written on the green forms. It is a nice practical point to remember that veronal requires a pink form to itself, but that malourea, the chemical equivalent, may be inscribed on the commoner background. In April next an important Special Number of *The Prescriber* will appear, devoted to hormone therapy.

